# Metagenomics of Antarctic Marine Sediment Reveals Potential for Diverse Chemolithoautotrophy

**DOI:** 10.1128/mSphere.00770-21

**Published:** 2021-11-24

**Authors:** Arkadiy I. Garber, Jessica R. Zehnpfennig, Cody S. Sheik, Michael W. Henson, Gustavo A. Ramírez, Andrew R. Mahon, Kenneth M. Halanych, Deric R. Learman

**Affiliations:** a Biodesign Center for Mechanisms for Evolution, Arizona State University, Tempe, Arizona, USA; b Department of Biology, Central Michigan University, Mt. Pleasant, Michigan, USA; c Biology Department and Large Lakes Observatory, University of Minnesota Duluth, Duluth, Minnesota, USA; d College of Veterinary Medicine, Western University of Health Sciences, Pomona, California, USA; e Department of Marine Biology, Haifa University, Haifa, Israel; f Center for Marine Science, University of North Carolina Wilmington, Wilmington, North Carolina, USA; National Institute of Advanced Industrial Science and Technology

**Keywords:** Antarctica, chemolithotrophy, marine microbiology, metagenomics, marine sediment

## Abstract

The microbial biogeochemical processes occurring in marine sediment in Antarctica remain underexplored due to limited access. Further, these polar habitats are unique, as they are being exposed to significant changes in their climate. To explore how microbes drive biogeochemistry in these sediments, we performed a shotgun metagenomic survey of marine surficial sediment (0 to 3 cm of the seafloor) collected from 13 locations in western Antarctica and assembled 16 high-quality metagenome assembled genomes for focused interrogation of the lifestyles of some abundant lineages. We observe an abundance of genes from pathways for the utilization of reduced carbon, sulfur, and nitrogen sources. Although organotrophy is pervasive, nitrification and sulfide oxidation are the dominant lithotrophic pathways and likely fuel carbon fixation via the reverse tricarboxylic acid and Calvin cycles. Oxygen-dependent terminal oxidases are common, and genes for reduction of oxidized nitrogen are sporadically present in our samples. Our results suggest that the underlying benthic communities are well primed for the utilization of settling organic matter, which is consistent with findings from highly productive surface water. Despite the genetic potential for nitrate reduction, the net catabolic pathway in our samples remains aerobic respiration, likely coupled to the oxidation of sulfur and nitrogen imported from the highly productive Antarctic water column above.

**IMPORTANCE** The impacts of climate change in polar regions, like Antarctica, have the potential to alter numerous ecosystems and biogeochemical cycles. Increasing temperature and freshwater runoff from melting ice can have profound impacts on the cycling of organic and inorganic nutrients between the pelagic and benthic ecosystems. Within the benthos, sediment microbial communities play a critical role in carbon mineralization and the cycles of essential nutrients like nitrogen and sulfur. Metagenomic data collected from sediment samples from the continental shelf of western Antarctica help to examine this unique system and document the metagenomic potential for lithotrophic metabolisms and the cycles of both nitrogen and sulfur, which support not only benthic microbes but also life in the pelagic zone.

## INTRODUCTION

Chemolithoautotrophic metabolisms play important roles in marine sediment ecosystems. For example, nitrification (oxidation of ammonia to nitrate) is considered an important process in global marine nitrogen cycles (1–4); however, little research has been done on ammonia oxidation in polar oceans (5, 6), especially for Antarctic benthic microbial community nitrification. A greater understanding of chemolithoautotrophic metabolisms is needed, as the cycling of nitrogen and sulfur not only drives microbial communities but also supports life in higher trophic levels in the Southern Ocean (7). Importantly, the biogeochemical cycling of nitrogen- and sulfur-containing compounds impacts the flux of elements relevant to benthic-pelagic coupling in an area being impacted by climate change.

Geochemical evidence suggests that nitrification is significant in polar regions, and chemoautotrophy, supported by nitrification, has been suggested as an important contribution to prokaryotic primary production during the polar winter (5, 8–12). Nitrification has been documented in Antarctic benthic sediments (13). Further, Franco et al. (14) found alphaproteobacterial operational taxonomic units (OTUs) that were similar to bacteria known to play a role in the nitrogen cycle. Learman et al. (15) documented an OTU related to *Thaumarchaeota*, an ammonium-oxidizing archaeon (AOA) commonly found in the euphotic water column (16) and marine sediments (1); this OTU was detected in western Antarctica regions with relatively low organic carbon (15). These ecological studies point to potential key microbial participants in the nitrogen cycle. However, direct examination of the genetic/functional potential for nitrification with this system is lacking.

The cycling of sulfur is also important for both lithotrophy and organic matter mineralization (17). In highly productive continental margin marine sediments, the sulfur cycle is driven by anaerobic microorganisms carrying out dissimilatory sulfate reduction (DSR) (17, 18), which acts as an important pathway for organic matter decomposition (17). Through a series of intermediates, DSR ultimately results in the formation of reduced sulfur. Sulfur (and its reduced intermediates, including thiosulfate and elemental sulfur) is an important source of energy in surface marine sediment due to availability of reduced sulfur compounds and oxygen (19–21), while in deeper anoxic sediment, sulfate reduction is more important (17, 22). Sulfur oxidation in Antarctica has been documented in subglacial outflow (23), lakes (24), and sediments (25). Sulfur oxidizers have also been found in surface sediment cores obtained from the Antarctica continental shelf within the Mertz Glacier polynya (26). Similar to nitrification, the genetic/functional potential for sulfur cycling in Antarctica marine sediments has not, to our knowledge, been directly investigated.

Previous microbial studies of Antarctic sediments have focused on how community diversity was impacted by organic matter (14, 15, 27–31). Western Antarctica, compared to the Antarctica Peninsula, has been shown to have lower concentrations of sedimentary organic matter (15). We hypothesize that this environmental condition is conducive to a diversity of lithotrophic metabolisms. To this end, we examined both shotgun metagenomic and geochemical data from surficial sediment samples from the continental shelf of western Antarctica (Amundsen Sea, Bellingshausen Sea, and Ross Sea) to document the genetic/functional potential for chemolithoautotrophy within this ecosystem. Our results provide insights into how microbes cycle nitrogen and sulfur and drive essential biogeochemical cycles in the Southern Ocean.

## RESULTS AND DISCUSSION

### Nitrification in benthic sediments.

**FIG 1:**
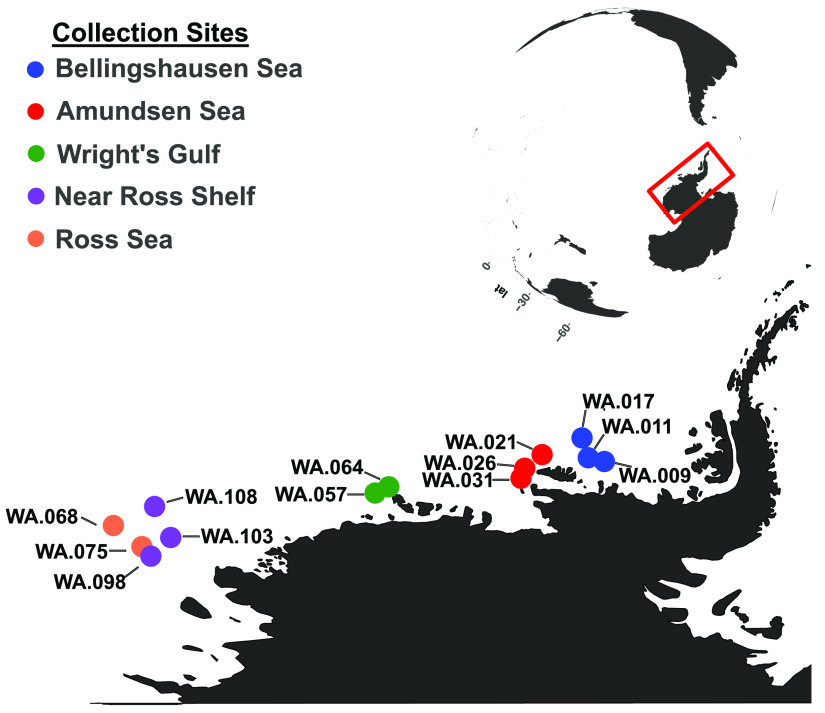
Map of Antarctica’s western peninsula, showing the geographic locations from which the metagenomic samples were derived.

**FIG 2:**
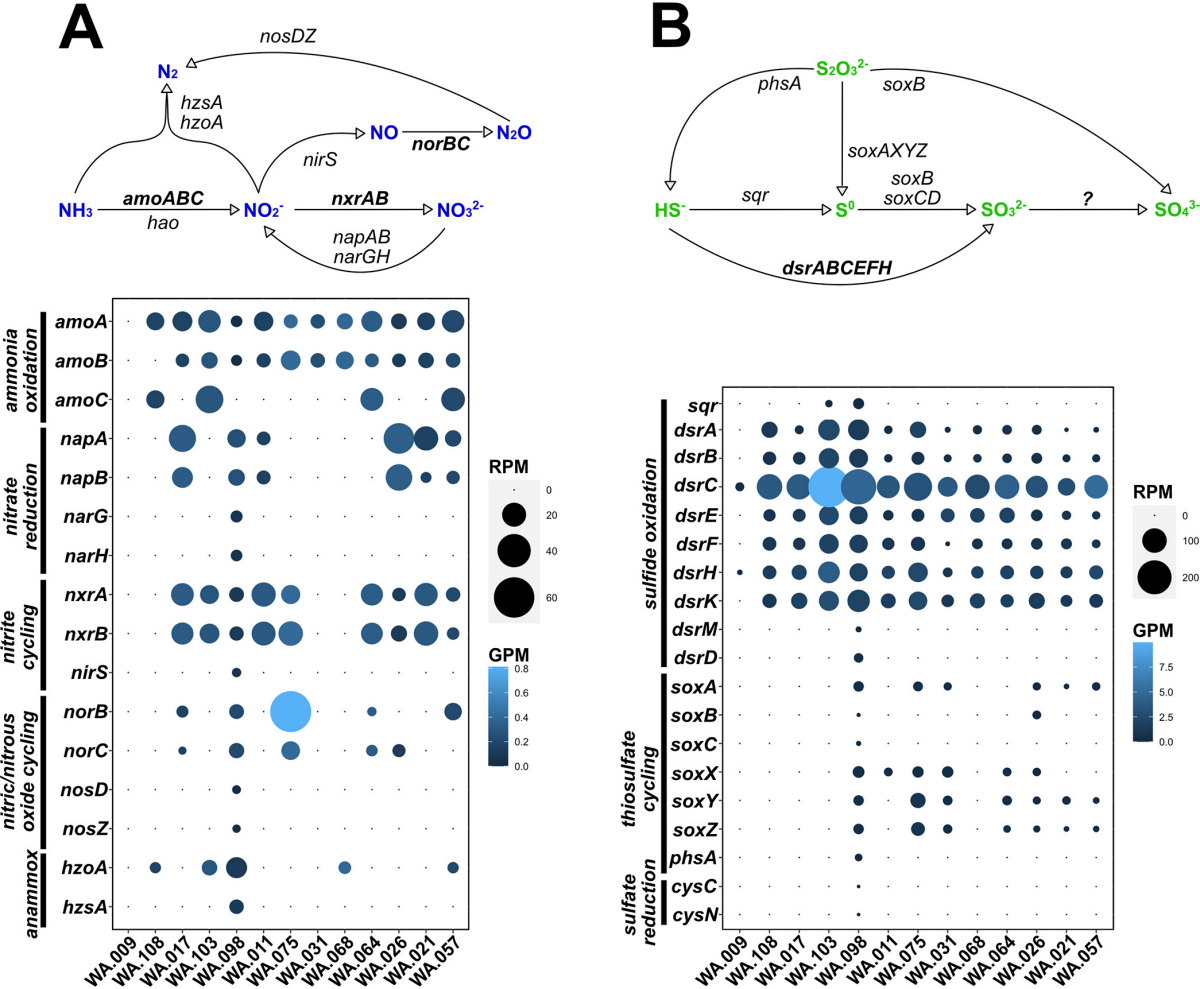
Dot plots and redox cycling of various nitrogen (A) and sulfur (B) compounds. The various elemental species are colored blue and green (for nitrogen and sulfur, respectively) to visually separate them from the names of genes attributed to each reaction. Genes that appear to be abundant or consistently present through all or most of the sites are shown in bold in the redox cycling schematics above each dot plot. The size of each dot represents reads per million (RPM), a measure of gene abundance based on gene mapping, normalized to the length of each gene and size of the data set. The color gradients denote genes per million (GPM), a measure of gene diversity based on the number of different gene homologs identified, normalized to the total number of genes predicted from each metagenome.

In 12 of the 13 western Antarctica (WA) sites spanning from the Ross Sea to the Amundsen Sea (Fig. 1 and 2A), we detected genes for ammonia oxidation (*amoABC*), the first step of the conversion of ammonia to nitrite (5). The identified *amo* genes were derived from *Nitrosomonas* and *Nitrosospira* (Table S1). This is consistent with previous reports of *Nitrosomonas* and *Nitrosospira* lineages in Antarctic surface waters (10), metabolizing ammonia and releasing nitrite as a by-product (32–36). The nitrite-oxidizing *nxrAB* genes, used previously as markers for nitrite-oxidizing bacteria (37–39), were also detected, but in only nine of the 12 sites that contain *amo* genes, supporting full nitrification of ammonia to nitrate in those sites (Fig. 2A). Phylogenetic comparisons of *nxrAB* genes reveal a close relationship to *Nitrospina* (Table S1), which is a known nitrite oxidizer in Antarctic marine sediments (40, 41) and coastal surface waters (10). The presence of both *amoABC* and *nxrAB* in sediments replete with oxygen (Table S2) (oxygen in the water above the sediments ranges from 4.1 to 6.2 mL/L) indicates that ammonia and nitrite are sources of energy in these communities.

10.1128/mSphere.00770-21.1 TABLE S1 Predicted metabolic pathways and their closest phylogenetic affiliations, based on their closest BLAST hits (E value < 1E−6) to NCBI’s RefSeq database. Download Table S1, DOCX file, 0.03 MB. Copyright © 2021 Garber et al. 2021 Garber et al. https://creativecommons.org/licenses/by/4.0/ This content is distributed under the terms of the Creative Commons Attribution 4.0 International license.

10.1128/mSphere.00770-21.2 TABLE S2 Sampling location and sediment nutrients. Download Table S2, DOCX file, 0.03 MB. Copyright © 2021 Garber et al. 2021 Garber et al. https://creativecommons.org/licenses/by/4.0/ This content is distributed under the terms of the Creative Commons Attribution 4.0 International license.

Unlike genes for ammonia and nitrite oxidation, genes for reductive nitrogen processes (e.g., nitrate and nitrite reduction) are less common throughout the 13 sequenced sites. For example, dissimilatory nitrate reductase genes, *napAB*, are found in six of the 13 sites, while genes for nitrous oxide reduction, *nosDZ*, are found in only one of the Antarctica sites. Even though it is found in only five of the 13 Antarctica site, in WA.075, the nitric oxide reduction gene *norB* displays the greatest copy number (∼60 reads per million [RPM], i.e., read coverage normalized to gene length and sequencing depth), as well as the greatest diversity (∼0.8 genes per million [GPM], i.e., number of different assembled *norB* sequences normalized to the total number of genes predicted from each site assembly). The gene *nirS*, encoding a nitrite reductase, however, appears to be absent from that assembly, so the source of nitric oxide in that site remains unknown.

**FIG 3:**
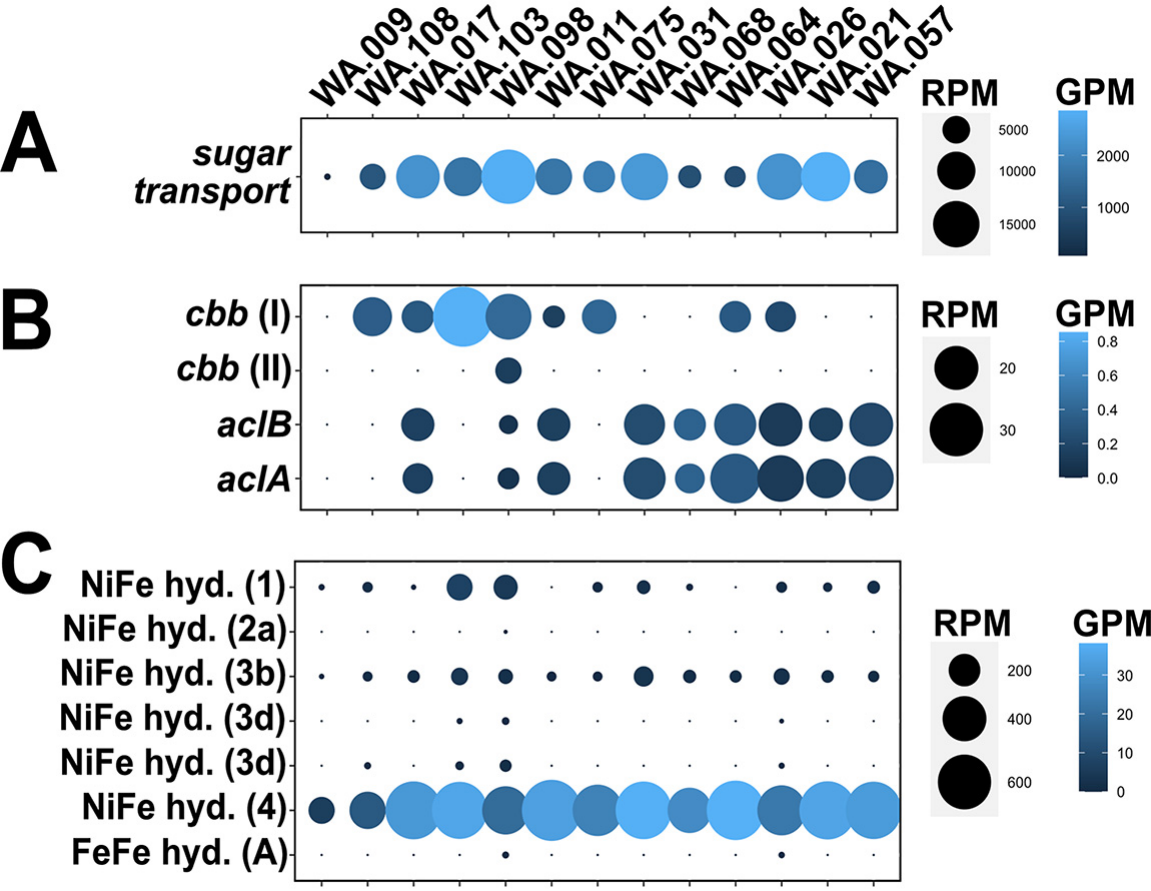
Dot plots for the sugar transporters (A), carbon fixation (B), and hydrogenases (hyd.) (C) identified in the metagenome assemblies. As in Fig. 2, the size of each dot represents reads per million (RPM), a measure of gene abundance based on gene mapping, normalized to the length of each gene and size of the data set. The color gradients denote genes per million (GPM), a measure of gene diversity based on the number of different gene homologs identified, normalized to the total number of genes predicted from each metagenome.

The most common pathways for nitrogen cycling, present in 12 of the 13 data sets, are those for oxidative reactions, converting ammonia to nitrate (i.e., nitrification). The absence of the full denitrification pathway in most assemblies could indicate that these sites were not sequenced deeply enough and the genes were not assembled or that the microbes responsible for the undetected parts of the denitrification pathway are part of the rare, or low-abundance, biosphere. In support of the latter possibility, WA.098, our most deeply sequenced site, appears to contain all required genes for the full nitrogen cycle. This sample, as well as four others, also contain *hzoA* and *hzsA* (Fig. 3), which are markers for anammox reactions (42, 43), allowing the direct conversion of ammonium to molecular nitrogen. Overall, during the southern summer, nitrification of reduced nitrogen sources, likely sourced from sinking detritus, is an important process that drives lithoautotrophy.

### Sulfur cycling.

In the benthic sediments along the western Antarctic Peninsula, genes encoding sulfate reduction are absent, with the exception of a single copy of a sulfate-reducing operon (*cys*) in site WA.098. Rather, the oxidative version of the dissimilatory sulfite reductase pathway (*rdsrAB*) was found in nearly all samples (Fig. 2B). Phylogenetic analysis of recovered *dsr* genes revealed their close relationship with those of *Gammaproteobacteria*, specifically, *Acidithiobacillales* and *Thioflexothrix* (Table S1). The additional presence of *dsrEFH*, genes specific to sulfur oxidation (39), suggests that the DSR pathway in these samples operates in reverse (i.e., reverse DSR) (44–48). This would suggest that sulfate is likely produced in the sampled sediments.

In contrast to the ubiquity of sulfide oxidation genes, genes for thiosulfate cycling via the *sox* and *phsA* pathways were found in only some of the sites. We did not detect the *soxCD* genes, which are required for complete oxidation of thiosulfate to sulfate (44). Rather, the absence of *soxCD* suggests that elemental sulfur may accumulate intracellularly and be oxidized to sulfate via the reverse DSR pathway and sulfide-quinone oxidoreductase (SQR) (Fig. 2B) (49–52). Our observations are consistent with previous reports of the mutual exclusivity of *dsr* and *soxCD* (53–55) reported for individual organisms. Here, we report this trend at the community (metagenome) level, with *soxCD* missing from all surveyed sites. Our data show that, in Antarctica benthic sediments, sulfur cycling is dominated by sulfide oxidation, which, in addition to ammonia oxidation, likely serves as a significant driver of lithoautotrophy.

### Organotrophy in Antarctica benthic sediment.

The genetic potential for organotrophy was also found in Antarctic sediments. Previous research suggested phytodetritus to be a large component of organic matter delivered to benthic sediments in coastal Antarctica (15). Indeed, we observe DNA sequences derived from cyanobacteria and eukaryotes, which we interpret as detrital factions (Fig. S1). All sites had comparable abundances of cyanobacterial contigs, with the exception of WA.009, which is consistent with the fact that this site had the lowest measured amount of total organic carbon (Table S1). Along with the presence of possible exogenous sources of carbon, we observed many genes coding for sugar transporters (Fig. 3A). These genes were more abundant (RPM and GPM), relative to other pathways (e.g., carbon fixation) (Fig. 3B). Using the CAZy database as a reference to search for complex carbohydrate metabolisms, we detected various genes, including those with carbohydrate-binding modules, as well as those encoding glycoside hydrolases and carbohydrate esterases (Fig. S2), all of which may be involved in chitin degradation. Complex organic matter, like chitin, is an important source of carbon and nitrogen in marine systems (56–60). Chitin is known to be sourced from crustaceans, such as krill (Euphausia superba) in Antarctica (61). Further, chitin-degrading (chitinolytic) bacteria have been documented in sediments from Antarctica (62, 63). Our identification of this genetic repertoire for the metabolism of complex carbon supports the fact that these benthic communities are primed to use detritus for energy and carbon.

10.1128/mSphere.00770-21.5 FIG S1 Bar plots showing the percent of contigs of (A) putatively cyanobacterial origin and (B) putatively eukaryotic origin from each metagenome assembly. Cyanobacterial contigs were identified as those where at least half of the top taxonomic hits (based on a DIAMOND BLAST search against proteins in NCBI’s nonredundant [nr] protein database) are to genes of cyanobacterial origin. Eukaryotic contigs were inferred from those which were >2 kb, which had a coding density <0.5 genes/kb, and for which more than half the hits were to proteins of eukaryotic origin. Download FIG S1, EPS file, 0.3 MB. Copyright © 2021 Garber et al. 2021 Garber et al. https://creativecommons.org/licenses/by/4.0/ This content is distributed under the terms of the Creative Commons Attribution 4.0 International license.

10.1128/mSphere.00770-21.6 FIG S2 Heat map of genes coding for carbohydrate-active enzymes, color coded to show which genes are known to be involved in the degradation of complex/recalcitrant carbon. Download FIG S2, EPS file, 0.7 MB. Copyright © 2021 Garber et al. 2021 Garber et al. https://creativecommons.org/licenses/by/4.0/ This content is distributed under the terms of the Creative Commons Attribution 4.0 International license.

The data also show hydrogen as an important energy source and by-product of the metabolic reactions occurring in these benthic sediments (Fig. 3C). By far, the most abundant hydrogenase detected is the hydrogen-evolving group 4 hydrogenase, which functions to relieve reducing equivalents generated by fermentation, conserving energy in the process (64, 65). Group 1 and 3b hydrogenases were also relatively ubiquitous among the sampled sediment. Group 3b hydrogenases are similar to group 4 in that they are coupled to fermentation, while group 1 hydrogenases allow the use of molecular hydrogen as an electron donor and source of energy (65). Indeed, fermentation, likely an important process in these sediments, can also serve as a source of hydrogen (64), which could be used for energy by other microbes. The ubiquitous presence of oxygen-dependent terminal oxidases, however, suggests that oxygen was present in our samples, inhibiting the efficiency of microbial fermentation.

The ubiquity and relative abundance of genes diagnostic of organotrophy suggest that benthic communities in Antarctica have the genetic potential to remineralize carbon. However, we also observe genes for carbon fixation (Fig. 3B). The *cbbI* (RuBisCO form 1) gene was found at eight sites and appears to be most common at WA.103. Genes involved in the reverse tricarboxylic acid (rTCA) cycle, another mechanism for carbon fixation, were present in nine sites, including sites where *cbbI* genes were not detected (with the exception of WA.009). We did not detect any genes for light sensing in our metagenomic data (e.g., proteorhodopsin or chlorophyll for photoheterotrophy and photoautotrophy). This is again consistent with the sediment depths from which these samples were collected (water depth range, 412 to 765 m). Thus, our detection of genes for carbon fixation (Fig. 3B) supports a role for chemolithoautotrophy, with reduced nitrogen and sulfur (Fig. S2) as the predominant sources of energy.

### Oxygen reduction.

Even though the sediment deposition rates at our samples sites are high (∼1 mm/year) (98), the surficial nature of our sediment samples, combined with the influence of bioturbation (13), implies that the bulk of the microbial constituents of the surveyed communities are exposed to oxic conditions. Accordingly, our detection of terminal oxidases (Fig. S3B) and relative dearth of genes diagnostic of anaerobic processes (e.g., nitrate and sulfate reduction) suggest that our sampled communities are poised to use oxygen as a final electron acceptor. Despite some signals suggesting the reduction of nitrate and, perhaps, iron (Fig. S3A), the net catabolic pathway in our samples remains aerobic respiration, likely coupled to organic matter oxidation.

10.1128/mSphere.00770-21.7 FIG S3 (A) Schematic for iron cycling and dot plots showing the presence/absence/abundance of genes for various reactions, including the redox cycling of iron (top dot plot) and iron acquisition/storage (bottom dot plot). (B) Dot plots for genes coding for terminal oxygen reductases. Download FIG S3, EPS file, 0.9 MB. Copyright © 2021 Garber et al. 2021 Garber et al. https://creativecommons.org/licenses/by/4.0/ This content is distributed under the terms of the Creative Commons Attribution 4.0 International license.

### Description of MAGs from the Ross Sea.

We recovered a total of 61 metagenome-assembled genomes (MAGs) from our deeply sequenced sample (WA.098); of these MAGs, 16 had completion scores above 60% (Table S3), and this subset of higher-quality MAGs was analyzed in more detail. The 16 MAGs ranged in size from 1.2 to 4.6 Mb, had GC contents ranging from 33% to 56%, and had genome completion scores between 61 and 99% (Table S3). Gene density ranged from 0.68 to 1.12 genes per kb (Fig. S4; Table S3). Gene density seemed to correlate inversely with MAG genome size (corrected using estimated completion scores) (Fig. S4). This rough correlation supports the idea that smaller, more-streamlined genomes encode less nongenic sequences, compared with larger genomes (66).

10.1128/mSphere.00770-21.3 TABLE S3 Assembly and annotation statistics. Download Table S3, DOCX file, 0.03 MB. Copyright © 2021 Garber et al. 2021 Garber et al. https://creativecommons.org/licenses/by/4.0/ This content is distributed under the terms of the Creative Commons Attribution 4.0 International license.

10.1128/mSphere.00770-21.8 FIG S4 Scatterplot showing the coding density (in genes/kilobase) of each of the 16 MAGs from WA.098, plotted against each MAGs estimated genome size (normalized to the completion of each MAG). The size of each dot represents the completeness score for each MAG, which, for 16 of the relatively complete MAGs, ranges from 99.3% to 61.2%. Download FIG S4, EPS file, 0.1 MB. Copyright © 2021 Garber et al. 2021 Garber et al. https://creativecommons.org/licenses/by/4.0/ This content is distributed under the terms of the Creative Commons Attribution 4.0 International license.

**FIG 4:**
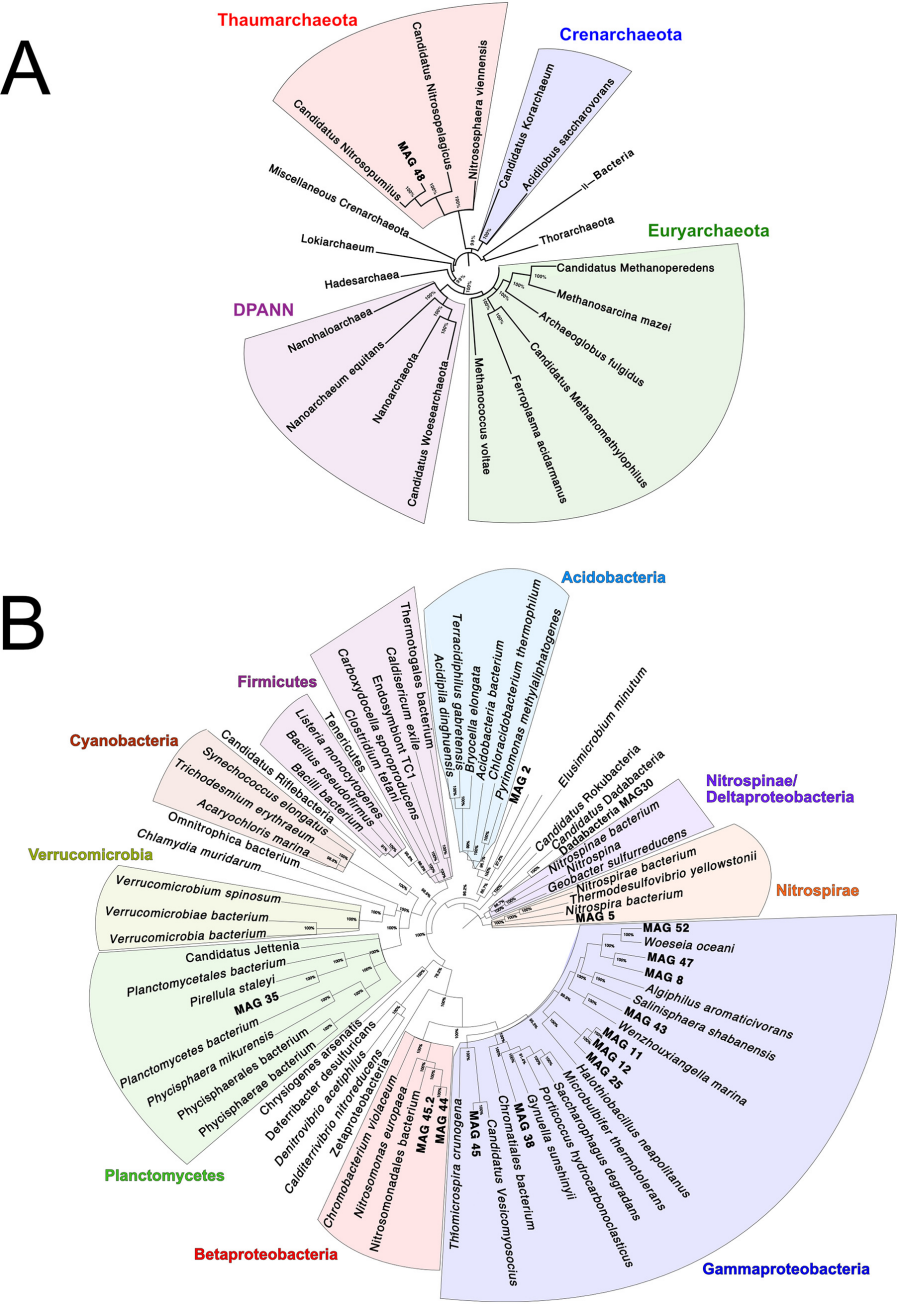
Phylogenomic trees demonstrating the taxonomic context for (A) one high-quality archaeal MAG and (B) 15 high-quality bacterial MAGs reconstructed from WA.098.

Only one archaeal MAG (MAG 48) was recovered, and is most closely related to *Nitrosopumilus*, within the phylum *Thaumarchaeota* (Fig. 4A), although analysis using the SprayNPray software (67) reveals that this MAG is only about 75% similar (average amino acid identity) to *Nitrosopumilus* sequences available in NCBI (Table S4). This thaumarchaeal MAG had one of the smallest estimated genome sizes (1.29 Mb) and the highest gene density (∼1.2 genes/kb), consistent with previous reports of streamlining in this lineage of *Archaea* (68). Thaumarchaea are generally considered to make a living by oxidizing ammonia (69), consistent with our broader metagenomic survey demonstrating ammonia oxidation as an important process in these Antarctica sediments. Although this MAG did not appear to harbor genes for ammonia oxidation, BLAST analysis revealed a three-gene operon, encoding hypothetical proteins, with remote homology to *amoABC*.

10.1128/mSphere.00770-21.4 TABLE S4 Summary information for each of the 16 high-quality MAGs generated from WA.098. Download Table S4, DOCX file, 0.03 MB. Copyright © 2021 Garber et al. 2021 Garber et al. https://creativecommons.org/licenses/by/4.0/ This content is distributed under the terms of the Creative Commons Attribution 4.0 International license.

**FIG 5:**
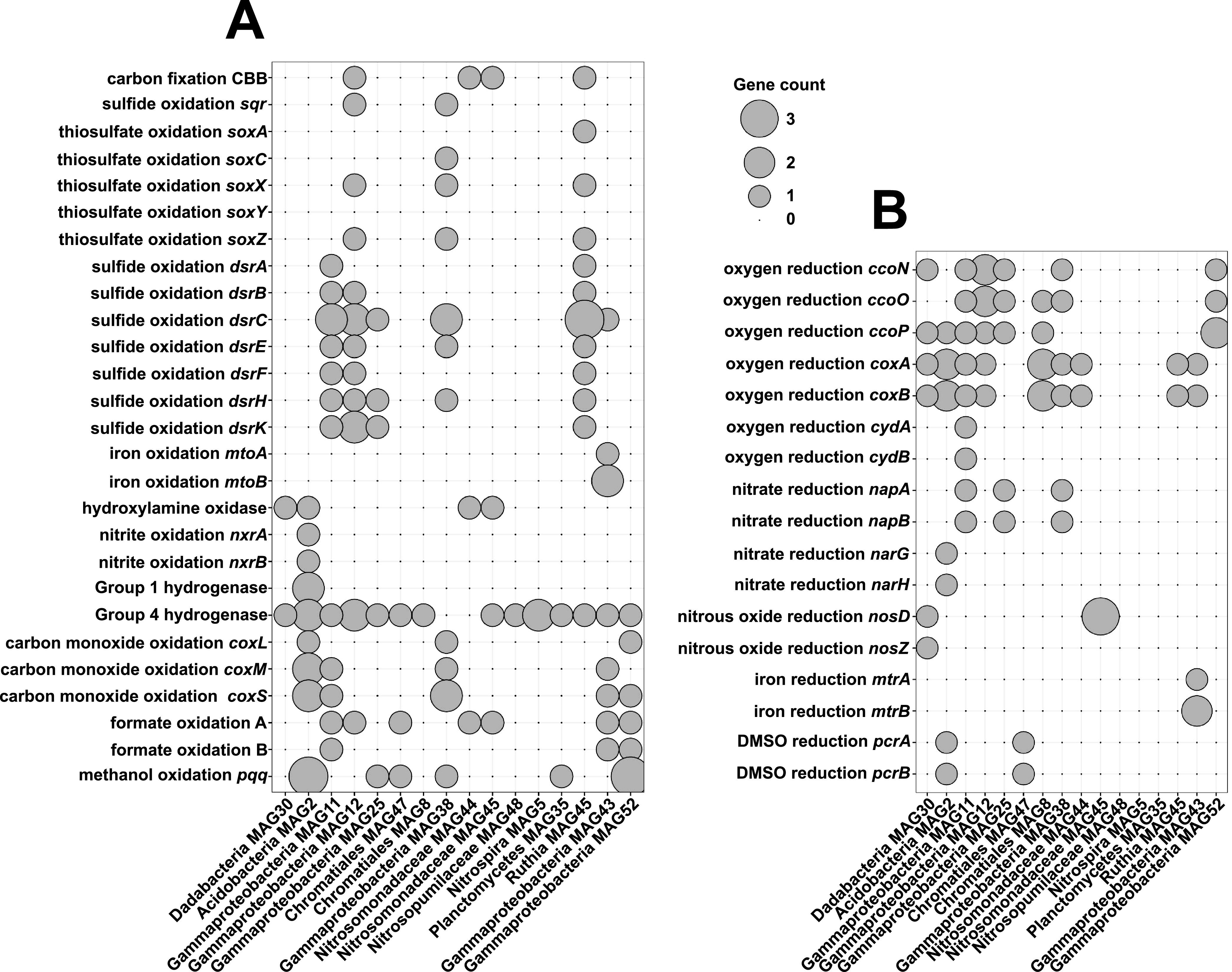
Dot plots summarizing (A) energy sources and (B) terminal electron acceptors utilized by the 16 high-quality MAGs from WA.098. DMSO, dimethyl sulfoxide.

Two of the MAGs (MAG 44 and MAG 45) were most closely related to the family *Nitrosomonadaceae*. This lineage is known to play a role in nitrification (32, 70) and may be drivers of the biogeochemical cycling of nitrogen that we observe in the nonbinned metagenome assemblies. However, we did not detect any ammonia oxidation genes (*amo*) genes in these MAGs (Fig. 5). These two *Nitrosomonadaceae* MAGs are 97% and 98% complete; the high genome completeness makes it relatively unlikely that *amo* genes are missing due to chance, although it is possible that the genes, which are generally highly abundant in our samples, ended up on short unbinned contigs. Nonetheless, both *Nitrosomonadaceae* MAGs harbor the hydroxylamine oxidase gene *hao*, allowing the oxidation of ammonium to nitrite. Oxidation of nitrite to nitrate could then be carried out by *Acidobacteria* MAG 2, which carries the nitrite oxidation genes *nxrAB*.

Four of the MAGs have the genetic potential to reduce nitrate via the dissimilatory nitrate reductase genes *napAB* and *narGH* (Fig. 5). Gammaproteobacteria MAG 11 has the genetic potential to reduce nitrite to nitric oxide. Although none of the 16 high-quality MAGs were found to carry genes for the reduction of nitric to nitrous oxide, the final step of denitrification (nitrous oxide reduction via *nosDZ*) is encoded by a MAG whose closest sequenced relative is part of the recently described phylum “*Candidatus* Dadabacteria.” This phylum is part of the candidate phyla radiation and has only been documented in marine ecosystems in the past 5 years (71, 72). Taken together, our data allowed us to identify seven MAGs that are likely key players in denitrification and may play key roles impacting nitrogen cycling in Antarctica sediment.

At least four MAGs from WA.098 harbor genes for sulfur cycling (Fig. 5). Three MAGs encode most of the DSR pathway for dissimilatory sulfite reduction. The presence of *dsrEFH* in these MAGs is indicative of reverse DSR, where sulfide is oxidized to sulfite. Two of these MAGs (MAG 11 and MAG 12) are within the *Gammaproteobacteria* but could not be further taxonomically resolved. One of the sulfur-oxidizing MAGs (MAG 45) appears to be affiliated with the genus “*Candidatus* Ruthia,” which consists of chemoautotrophic sulfur-oxidizing symbionts (73), sharing ∼86% average amino acid identity with the sequenced “*Candidatus* Ruthia endofausta” genome. Similar to the genome sequence of “*Candidatus* Ruthia magnifica,” a symbiont of a hydrothermal vent clam (73, 74), the 99.3%-complete MAG 45 has a 1.3-Mbp genome, a relatively small size for bacteria (very similar in size to the recovered thaumarchaea-related MAG [MAG 48]), and a low GC content (37.3%). Both of these properties are hallmarks of endosymbionts having undergone genome reduction (75). The chemoautotrophic symbiont MAG 45 also has genes for thiosulfate oxidation (*soxZXA*), which provides additional energy for carbon fixation via the Calvin-Benson-Bassham (CBB) cycle.

The endosymbiont MAG 45 likely carries out carbon fixation inside a host, but three other MAGs, presumably free-living, were also found to encode the CBB carbon fixation pathway (Fig. 5). One of these MAGs (*Gammaproteobacteria* MAG 12) also has genes for sulfide and thiosulfate oxidation, via reverse DSR and *soxZX*, respectively. The other two MAGs that encode carbon fixation pathways (*Nitrosomonadaceae* MAG 44 and MAG 45) are affiliated with known ammonia oxidizers but, as mentioned above, do not appear to have genes for ammonia oxidation (Fig. 5).

This study was not able to assign any MAGs to a specific genus. For example, one bin clustered with Pirellula staleyi but shares only about 60% amino acid identity with closest homologs available in NCBI (and even less with the reference *P. staleyi* genome). Analysis using the SprayNPray (62) software reveals that in most of the 16 bins examined, the taxonomic affiliations of the top hits to each bin are from an unexpectedly high diversity of species, genera, and, in some cases, phyla (Fig. S5). Notably, differences in top taxonomic hits were found not only between contigs but also between different regions within individual contigs. These observations could be the result of chimeric assemblies (76) or, more likely, due to poor representation of the sequenced microorganisms in NCBI’s nonredundant (nr) database. By comparing the average amino acid identities with the number of different taxonomic hits to each bin, we observe an inverse relationship between these two variables, where MAGs with higher amino acid identity to representative orthologs in NCBI have a significantly (*P* = 1.254e−06) lower variety of taxonomic hits (Fig. S6). Some of this taxonomic diversity is possibly the result of pervasive horizontal gene transfer events. For example, three of the MAGs that clustered within the class *Gammaproteobacteria* (*Gammaproteobacteria* MAG 11, MAG 12, and MAG 25) recruit hits from a wide variety of taxa, but mostly within the phylum *Proteobacteria*; however, some of the proteobacterial contigs also recruit hits to *Planctomycetes*, *Chloroflexi*, and *Firmicutes* genes. While it is possible that some of these discordant DIAMOND hits may represent horizontal gene transfer (HGT) events, it is unlikely that all of the observed taxonomic inconsistency is due to HGT alone.

10.1128/mSphere.00770-21.9 FIG S5 Word clouds representing the top DIAMOND BLAST hits (from NCBI’s nr database) to each of the 16 relatively complete MAGs from WA.098. The MAG designation, along with the average amino acid identity (AAI) to the top DIAMOND BLAST hits from the nr database, are shown in green. Download FIG S5, EPS file, 0.9 MB. Copyright © 2021 Garber et al. 2021 Garber et al. https://creativecommons.org/licenses/by/4.0/ This content is distributed under the terms of the Creative Commons Attribution 4.0 International license.

10.1128/mSphere.00770-21.10 FIG S6 Scatterplot showing the number of different taxonomic DIAMOND BLAST hits recruited to each MAG from the nr database, as a function of the average amino acid identity to those top DIAMOND BLAST hits. This shows that the more closely a genome is related to sequences in NCBI, the more consistent (less random) are the top taxonomic hits, resulting in a more reliable and stable taxonomic positioning. Download FIG S6, EPS file, 0.1 MB. Copyright © 2021 Garber et al. 2021 Garber et al. https://creativecommons.org/licenses/by/4.0/ This content is distributed under the terms of the Creative Commons Attribution 4.0 International license.

### Conclusions.

Overall, our results reveal sedimentary communities that benefit from the input of reduced nitrogen, sulfur, and carbon, likely from the overlying water column. Genetic potential for lithotrophic metabolism was abundantly documented in sediments from the Ross to the Bellingshausen Sea. Most of the MAGs defined in the Ross Sea were not able to be placed into a specific genus, relative to what is known in published databases, which suggests that this ecosystem hosts organisms that are unique and novel. Nonetheless, these MAGs from Ross Sea, as well as the functional potential observed in all of our sequenced samples, show that these communities may play key roles in the pelagic-benthic biogeochemical cycling of important compounds in Southern Ocean waters off western Antarctica.

## MATERIALS AND METHODS

### Sampling details.

Surface sediment samples from the continental shelf of western Antarctica (WA) were collected on the *RVIB Nathaniel B. Palmer* (December 2013 to February 2014) using a MC-800 multicorer (Ocean Instruments). Surface sediment from the top of the cores (approximately the top 3 cm) was aseptically transferred with a spatula into conical tubes and immediately frozen (−80°C). Samples were shipped frozen from the field after collection to the lab at Central Michigan University (CMU). The sampling locations, which include the Amundsen Sea, Bellingshausen Sea, and Ross Sea (Fig. 1), have low organic matter relative to the Antarctica Peninsula (15), and as the sediments were sampled in the austral summer, they were at the forefront of incoming carbon flux from the surface waters. A detailed account of sampling locations and sediment nutrient data was published previously (15), and an abbreviated list can be found in Table S1.

### DNA extraction and sequencing.

DNA from sediment was extracted and cleaned as previously reported by Learman et al. (15). Briefly, DNA was extracted using a PowerSoil DNA extraction kit (MoBio) and concentrated using a DNA Clean & Concentrator kit (Zymo). Clean and concentrated DNA was quantified using a Qubit2.0 fluorometer (Life Technologies) and stored at −20°C. DNA for shotgun metagenomics was sequenced using an Illumina HiSeq 2500 instrument with paired-end 150-bp reads at Michigan State University’s Research Technology Support Facility (RTSF) Genomics Core.

### Assembly and binning.

Raw reads were initially checked for quality with FastQC (https://www.bioinformatics.babraham.ac.uk/projects/fastqc/) and trimmed with Trimmomatic v0.33 using default parameters (77). Reads were then assembled with metaSPAdes v3.15.2 (78). The sample from WA.098 was randomly chosen as a representative sample for the data set and was sequenced more deeply to support the assembly of metagenome-assembled genomes (MAGs) (Table S2). The resulting SPAdes assembly from WA.098 was then binned into MAGs with Metabat2 (79) using multiple parameters (superspecific, veryspecific, specific, sensitive, and verysensitive). The resulting MAGs were then consolidated using DASTool (80), and manually curated using Anvi’o v5 (81). The final completion and redundancy scores of the resulting MAGs were calculated using Anvi’o (Table S3).

### Annotation.

All assemblies were annotated using the DOE Joint Genome Institutes (JGI) Integrated Microbial Genomes (IMG) (82, 83) and Prodigal v/2.6.3 (84). IMG GOLD genome ID numbers can be found in Table S3 and S4. Shotgun metagenomic assemblies recovered 15,085 to 188,505 contigs of >1,000 bp (Table S2), resulting in 325,721 (WA.068) to 2,486,981 (WA.098) protein-coding genes (defined by IMG annotations) (Table S4). To further target lithotrophic metabolisms, site assemblies and MAGs were additionally annotated using MagicLamp (https://github.com/Arkadiy-Garber/MagicLamp). This software uses a set of publicly available HMMs, designed and compiled from Pfam and TIGRFAMS (85). We also used FeGenie to identify genes relevant to iron cycling (86). To target genes associated with recalcitrant carbon degradation, we used a set of KEGG Orthology identifiers published by Anantharaman et al. (85). Carbohydrate-active enzymes were identified using the CAZy database (87). Moreover, we used GhostKOALA (88) to generate KEGG Orthology identifiers for genes predicted from our data sets; KEGG-Decoder (89) was used to organize the KEGG Orthology annotation data into KEGG module pathways based on percent completion.

### Phylogenetic placement.

Using GToTree (90), we generated phylogenomic trees of high-quality WA.098 MAGs (completion > 60%; contamination < 8.7%). In addition to the MAGs collected herein, for taxonomic context, we included a phylogenetically broad set of genomes downloaded from RefSeq (91). To assess the evolutionary placement of the archaeal MAG, we used the *Archaea*-specific single-copy gene (SCG) set that is available within the GToTree package (90). For the rest of the MAGs that were within the domain *Bacteria*, we used the *Bacteria*-specific SCG set. Trees were visualized using FigTree (http://tree.bio.ed.ac.uk/software/figtree/). Taxonomic assignment to MAGs was done using GTDB-Tk, which uses a combination of metrics, including the average nucleotide identity to reference genomes in the NCBI Assembly database, placement in the GTDB reference tree, and the relative evolutionary divergence (92, 93).

To better understand the taxonomic relationship of the high-quality MAGs from WA.098 to previously sequenced organism, we used SprayNPray (https://github.com/Arkadiy-Garber/SprayNPray), which queries, using DIAMOND (94), the genes from each MAG against a reference database (NCBI nonredundant proteins [nr]). DIAMOND results are then parsed and visually inspected for downstream processing (Table S3).

Finally, we examined the phylogenetic placement of the *dsr*, *amo*, and *cyc2* genes as a proxy for determining function. Using BLAST (95), we identified homologs to select genes in RefSeq (91), with alignments generated with Muscle (96). Subsequently, phylogenetic trees were then generated using RAxML (substitution matrix = PROTCATBLOSUM62) (97) and visualized with FigTree.

### Data availability.

Raw sequencing reads were deposited in the NCBI Sequence Read Archive (SRA) under the project number PRJNA573088.
