# Supplementary material for: Metagenomics of Antarctic Marine Sediment Reveals Potential for Diverse Chemolithoautotrophy
Source: mSphere. 2021 Nov 24;6(6):e00770-21. doi: 10.1128/mSphere.00770-21 (PMC8612310; doi:10.1128/mSphere.00770-21)
Supplement: TABLE S1 [file msphere.00770-21-st001.docx]

**Table S1**. Predicted metabolic pathways and their closes phylogenetic affiliations, based on their closest BLAST hits (e-value < 1E-6) to NCBI’s RefSeq database.

| ***Metagenome Sample*** | ***Reaction*** | ***Genetic Markers*** | ***Closest Taxonomic Affiliation*** |
| --- | --- | --- | --- |
| WA.009 | hydrogen oxidation | Hydrogenase Group 4 | Chromatiales |
| WA.011 | ammonia oxidation | *amoABC* | *Nitrosomonas* |
|  | carbon fixation (CCB) | *rubisco form 1* | *Nitrosospira* sp. |
|  | carbon fixation (rTCA) | *aclAB* | *Nitrospira* bacterium SG8_3 |
|  | hydrogen oxidation | Hydrogenase Group 4 | Chromatiales |
|  | iron oxidation | *cyc2* (cluster III) | Acidobacteria |
|  | thiosulfate oxidation | *soxXYZ* | Rhodospirillaceae |
| WA.017 | ammonia oxidation | *amoAC* | *Nitrosospira* |
|  | carbon fixation (CCB) | rubisco form 1 | *Nitrosospira* |
|  | carbon fixation (rTCA) | *aclAB* | Nitrospira bacterium SG8_3 |
|  | hydrogen oxidation | hydrogenase group 4 | Acidithiobacillales bacterium SG8_45 |
|  |  |  | *Woeseia oceani* |
|  | iron oxidation | *cyc2* (cluster III) | Acidobacteria |
|  | nitrite oxidation | *nxrAB* | *Nitrospina* |
|  | thiosulfate oxidation | *soxXYZ* | Rhodobacteraceae |
| WA.021 | nitrite oxidation | *nxrAB* | *Nitrospina* |
|  | hydrogen oxidation | hydrogenase group 4 | Acidithiobacillales bacterium SG8_45 |
|  |  |  | *Woeseia oceani* |
| WA.026 | ammonia oxidation | *amoABC* | *Nitrosospira* |
|  | carbon fixation (CCB) | rubisco form 1 | *Nitrosospira* |
|  | carbon fixation (rTCA) | *aclAB* | *Nitrospira* bacterium SG8_3 |
|  | iron reduction | *omcS* | Candidate Division Zixibacteria |
|  | hydrogen oxidation | hydrogenase group 4 | Acidithiobacillales bacterium SG8_45 |
|  |  |  | Chromatiales |
|  | iron oxidation | *sulfocyanin* | *Verrucomicrobium* |
|  | sulfite reduction/sulfur oxidation | *(r)dsrBCEFH* | Gammaproteobacteria |
| WA.031 | ammonia oxidation | *amoAB* | *Nitrosospira* |
|  | carbon fixation (rTCA) | *aclAB* | *Nitrospira* bacterium SG8_3 |
|  | hydrogen oxidation | hydrogenase group 4 | Acidithiobacillales bacterium SG8_45 |
|  |  |  | *Woeseia oceani* |
|  | iron reduction | omcS | Candidate Division Zixibacteria |
|  | thiosulfate oxidation | *soxAXYZ* | Rhodospirillaceae |
| WA.057 | hydrogen oxidation | Hydrogenase Group 4 | *Woeseia oceani* |
| WA.064 | hydrogen oxidation | Hydrogenase Group 4 | Acidithiobacillales bacterium SG8_45 |
|  | carbon fixation (CCB) | rubisco form 1 | Gammaproteobacteria incertae sedis (sulfur-oxidizing symbionts) |
|  | carbon fixation (rTCA) | *aclAB* | *Nitrospira* bacterium SG8_3 |
| WA.068 | ammonia oxidation | *amoAB* | *Nitrosospira* |
|  | carbon fixation (rTCA) | *aclAB* | *Nitrospira* bacterium SG8_3 |
| WA.075 | ammonia oxidation | *amoAB* | *Nitrosospira* |
|  | hydrogen oxidation | Hydrogenase Group 4 | Acidithiobacillales bacterium SG8_45 |
|  | iron oxidation | sulfocyanin | Verrucomicrobia |
|  | thiosulfate oxidation | *soxABYZ* | Alphaproteobacteria |
| WA.098 | ammonia oxidation | *amoABC* | *Nitrosospira* |
|  | carbon fixation (CCB) | rubisco form 1 | Gammaproteobacteria incertae sedis (sulfur-oxidizing symbionts) |
|  |  |  | *Nitrosospira* |
|  | carbon fixation (rTCA) | *aclAB* | *Nitrospira* bacterium SG8_3 |
|  | hydrogen oxidation | hydrogenase group 4 | Acidithiobacillales bacterium SG8_45 |
|  |  |  | *Woeseia oceani* |
|  | iron oxidation | sulfocyanin | Caldilineae |
|  |  |  | *Verrucomicrobium* |
|  | iron oxidation | *mtoAB* | Unclassified |
|  | iron reduction | *omcS* | *Geoalkalibacter* |
|  |  |  | Nitrospiraceae |
|  |  |  | *Desulfuromonas* |
|  | nitrite oxidation | *nxrAB* | Acidobacteria |
|  |  | *mtrAB* | Gammaproteobacteria |
|  | sulfide oxidation/detoxification | *sqr* | *Magnetovibrio* |
|  |  |  | *Myxococcales* |
|  |  |  | Gammaproteobacteria |
|  | sulfite reduction/sulfur oxidation | *dsrABCEFGHK* | Gammaproteobacteria |
|  | thiosulfate oxidation | *soxABXYZ* | Gammaproteobacteria |
|  |  |  | Rhodospirillaceae |
| WA.103 | ammonia oxidation | *amoAB* | *Nitrosospira* |
|  | carbon fixation (CCB) | rubisco form 1 | Gammaproteobacteria incertae sedis (sulfur-oxidizing symbionts) |
|  | hydrogen oxidation | Hydrogenase Group 4 | *Woeseia oceani* |
|  | iron oxidation | *mtoAB* | Unclassified |
|  | nitrite oxidation | *nxrAB* | *Nitrospina* |
|  | sulfite reduction/sulfur oxidation | *dsrABCEFH* | Gammaproteobacteria |
|  |  |  |  |
| WA.108 | ammonia oxidation | *amoABC* | *Nitrosospira* |
|  | carbon fixation (CCB) | rubisco form 1 | Gammaproteobacteria incertae sedis (sulfur-oxidizing symbionts) |
|  | hydrogen oxidation | Hydrogenase Group 4 | Chromatiales |
