# Supplementary material for: Metagenomics of Antarctic Marine Sediment Reveals Potential for Diverse Chemolithoautotrophy
Source: mSphere. 2021 Nov 24;6(6):e00770-21. doi: 10.1128/mSphere.00770-21 (PMC8612310; doi:10.1128/mSphere.00770-21)
Supplement: TABLE S2 [file msphere.00770-21-st002.docx]

**Table S2. Sampling location and sediment nutrients**

| **Site Names** | **Basin** | **Water Depth (m)** | **pH^*^** | **Total Organic Carbon (%)** | **Nitrogen (%)** | **d13C (TOC)** | **d15N** | **C/N** | **NH_4_-N (mg/kg)** | **NO_3_-N (mg/kg)** | **Sulfur (mg/kg)** | **Oxygen**  **SBE 43 (ml/l)*** |
| --- | --- | --- | --- | --- | --- | --- | --- | --- | --- | --- | --- | --- |
| WA.009 | Bellingshausen Sea | 412 | 7.4 | 0.13 | 0.02 | -24.88 | DL | 7.7 | 5.4 | 0.30 | 550 | 4.1 |
| WA.011 | Bellingshausen Sea | 672 | 7.4 | 0.43 | 0.05 | -24.27 | 2.60 | 9.9 | 4.9 | 0.42 | 551 | 4.2 |
| WA.017 | Bellingshausen Sea | 476 | 7.4 | 0.38 | 0.05 | -25.49 | 1.21 | 9.3 | 4.9 | 0.62 | 498 | 4.2 |
| WA.021 | Amundsen Sea | 471 | 7.3 | 0.35 | 0.04 | -26.30 | DL | 9.2 | 4.4 | 0.45 | 216 | 5.1 |
| WA.026 | Amundsen Sea | 597 | 7.3 | 0.52 | 0.09 | -25.74 | 3.43 | 6.8 | 4.4 | 0.32 | 573 | 5.2 |
| WA.031 | Amundsen Sea | 572 | 7.4 | 0.55 | 0.08 | -25.89 | 3.45 | 7.7 | 4.6 | 0.27 | 574 | 5.2 |
| WA.057 | Wright’s Gulf | 510 | 7.2 | 0.63 | 0.10 | -24.78 | 4.06 | 7.5 | 6.7 | 0.30 | 307 | 5.9 |
| WA.064 | Wright’s Gulf | 478 | 7.3 | 0.57 | 0.08 | -24.80 | 4.13 | 8.1 | 7.3 | 1.40 | 604 | 6.3 |
| WA.068 | Ross Sea | 567 | 7.5 | 0.32 | 0.04 | -26.39 | DL | 10.4 | 6.6 | 0.50 | 163 | 6.3 |
| WA.075 | Ross Sea | 531 | 7.3 | 0.44 | 0.06 | -26.18 | 1.97 | 8.2 | 6.8 | 0.34 | 443 | 6.5 |
| WA.098 | Near Ross Shelf | 765 | 7.3 | 0.69 | 0.09 | -27.49 | 2.13 | 8.9 | 7.3 | 0.45 | 187 | 6.2 |
| WA.103 | Near Ross Shelf | 552 | 7.3 | 0.85 | 0.13 | -24.41 | 3.02 | 7.6 | 5.6 | 0.30 | 490 | 6.2 |
| WA.108 | Near Ross Shelf | 528 | 7.2 | 0.86 | 0.13 | -25.05 | 3.04 | 7.4 | 6.6 | 0.29 | 621 | 6.2 |

*data taken from the CTD near the sediments

**DL denotes the value was below the detection limited
