# Supplementary material for: Metagenomics of Antarctic Marine Sediment Reveals Potential for Diverse Chemolithoautotrophy
Source: mSphere. 2021 Nov 24;6(6):e00770-21. doi: 10.1128/mSphere.00770-21 (PMC8612310; doi:10.1128/mSphere.00770-21)
Supplement: TABLE S3 [file msphere.00770-21-st003.docx]

**Table S3. Assembly and Annotation statistics**

|  | **Read Pairs** | **Number of Sequences** | **Average Sequence Length** | **Sequences over 300 bp** | **Sequences over 1000 bp** | **Sequences over 10000 bp** | **Protein coding genes** | **Protein coding genes with product name** | **COG Clusters** | **IMG Genome ID** |
| --- | --- | --- | --- | --- | --- | --- | --- | --- | --- | --- |
| WA.009 | 9,842,069 | 837,898 | 453 | 837,898 | 21,077 | 37 | 535,938 | 59,062 | 4457 | 3300034971 |
| WA.011 | 10,534,294 | 760,702 | 468 | 760,702 | 29,928 | 108 | 791,925 | 434,176 | 4547 | 3300034970 |
| WA.017 | 11,722,348 | 694,627 | 471 | 694,627 | 30,237 | 38 | 771,648 | 426,361 | 4531 | 3300034973 |
| WA.021 | 11,829,647 | 814,822 | 463 | 814,822 | 31,290 | 42 | 826,737 | 448,449 | 4542 | 3300034978 |
| WA.026 | 11,630,244 | 795,093 | 503 | 795,093 | 43,787 | 179 | 841,465 | 430,092 | 4564 | 3300034977 |
| WA.031 | 7,893,987 | 538,084 | 438 | 538,084 | 13,779 | 2 | 546,430 | 301,196 | 4438 | 3300034969 |
| WA.057 | 9,431,835 | 603,120 | 469 | 603,120 | 24,276 | 91 | 614,797 | 340,791 | 4511 | 3300034979 |
| WA.064 | 7,315,251 | 394,006 | 466 | 394,006 | 15,968 | 32 | 383,929 | 196,419 | 4408 | 3300034976 |
| WA.068 | 7,653,165 | 351,387 | 448 | 351,387 | 11,269 | 5 | 325,721 | 139,830 | 4438 | 3300034975 |
| WA.075 | 9,192,724 | 174,635 | 655 | 174,635 | 17,033 | 23 | 476,137 | 212,860 | 4559 | 3300034968 |
| WA.098 | 28,646,077 | 2,142,248 | 552 | 2,142,248 | 163,966 | 1,007 | 2,486,981 | 1,308,011 | 4626 | 3300035196 |
| WA.103 | 7,405,176 | 451,535 | 468 | 451,535 | 17,427 | 73 | 451,797 | 257,222 | 4426 | 3300034974 |
| WA.108 | 11,852,518 | 562,288 | 461 | 562,288 | 21,132 | 57 | 524,334 | 176,519 | 4514 | 3300034972 |
