# Supplementary material for: Metagenomics of Antarctic Marine Sediment Reveals Potential for Diverse Chemolithoautotrophy
Source: mSphere. 2021 Nov 24;6(6):e00770-21. doi: 10.1128/mSphere.00770-21 (PMC8612310; doi:10.1128/mSphere.00770-21)
Supplement: TABLE S4 [file msphere.00770-21-st004.docx]

**Table S4. Summary information for each of the 16 high-quality MAGs that were generated from WA.098**

| **Genome Bin** | **MAG Size (Mb)** | **GC %** |  |  |  |  | **Gene Count** | **Protein coding genes with function prediction** | **Comp. (%)^a^** |  | **Red Value^c^** | **IMG Genome ID** |
| --- | --- | --- | --- | --- | --- | --- | --- | --- | --- | --- | --- | --- |
|  |  |  | **Number of contigs** | **Average length of contigs** | **Genes per kb** | **Average percent AAI to NCBI nr proteins** |  |  |  | **Redun. or Contam. (%)^b^** |  |  |
| *Nitrosopumilus* *sp*. (MAG 48) | 1.29 | 33% | 163 | 7925 | 1.119 | 75 | 1736 | 1115 | 80.9 | 6.2 | 0.9 | 2828531757 |
| Candidatus Dadabacteria bacterium (MAG 30) | 2.28 | 40% | 271 | 8407 | 0.818 | 62 | 2464 | 1790 | 93.5 | 5.8 | N/A | 2828569208 |
| Acidobacteria (MAG 2) | 3.42 | 54% | 469 | 7255 | 0.731 | 52 | 3342 | 2312 | 93.5 | 1.4 | 0.76 | 2828565865 |
| Chromatiales bacterium (MAG 11) | 3.28 | 53% | 418 | 8086 | 0.859 | 59 | 3567 | 2700 | 66.9 | 0.7 | 0.94 | 2828541481 |
| Chromatiales bacterium (MAG 12) | 2.91 | 53% | 624 | 4666 | 0.897 | 60 | 3138 | 2490 | 69.1 | 3.6 | 0.94 | 2828538342 |
| Chromatiales bacterium (MAG 25) | 2.7 | 54% | 565 | 4759 | 0.841 | 59 | 2727 | 2120 | 76.3 | 2.9 | 0.94 | 2828535614 |
| Gammaproteobacteria bacterium (MAG 47) | 2 | 55% | 317 | 6303 | 0.851 | 57 | 2169 | 1648 | 87.1 | 6.5 | 0.87 | 2828529587 |
| Gammaproteobacteria bacterium (MAG 8) | 1.88 | 52% | 358 | 5263 | 0.876 | 57 | 2119 | 1605 | 80.6 | 5 | 0.9 | 2828533494 |
| *Gynuella* *sp*. (MAG 38) | 3.57 | 49% | 884 | 4022 | 0.877 | 59 | 4032 | 2892 | 78.4 | 5.8 | 0.92 | 2828554611 |
| *Nitrosomonas* *sp*. (MAG 44) | 1.94 | 43% | 205 | 9427 | 0.845 | 69 | 2055 | 1557 | 97.1 | 3.6 | 0.98 | 2828549778 |
| *Nitrosomonas* *sp*. (MAG 45) | 1.9 | 41% | 146 | 12877 | 0.835 | 67 | 1965 | 1502 | 97.8 | 0.7 | 0.95 | 2828518009 |
| Nitrospirae bacterium (MAG 5) | 1.26 | 49% | 317 | 3968 | 0.915 | 66 | 1474 | 1033 | 61.2 | 0.7 | 0.95 | 2828545049 |
| *Pirellula* *sp*. (MAG 35) | 4.58 | 55% | 1092 | 4081 | 0.681 | 55 | 4645 | 2501 | 70.5 | 2.9 | 0.82 | 2828558644 |
| Proteobacteria bacterium (MAG 45) | 1.31 | 37% | 87 | 15078 | 0.915 | 83 | 1481 | 1130 | 99.3 | 1.4 | 0.96 | 2828519975 |
| *Wenzhouxiangella* *sp*. (MAG 43) | 2.61 | 56% | 497 | 5254 | 0.856 | 57 | 2776 | 2124 | 90.6 | 0 | 0.83 | 2828551834 |
| *Woeseia* *sp*. (MAG 52) | 2.39 | 56% | 454 | 5260 | 0.921 | 64 | 2574 | 1998 | 67.6 | 0.7 | 0.95 | 2828563290 |

**^a^ Comp: Completion**

**^b^ Redun: Redundancy**

^c^ **RED: Relative Evolutionary Divergence (from GTdB Tk)**
